# Evaluation of Presumably Disease Causing *SCN1A* Variants in a Cohort of Common Epilepsy Syndromes

**DOI:** 10.1371/journal.pone.0150426

**Published:** 2016-03-18

**Authors:** Dennis Lal, Eva M. Reinthaler, Borislav Dejanovic, Patrick May, Holger Thiele, Anna-Elina Lehesjoki, Günter Schwarz, Erik Riesch, M. Arfan Ikram, Cornelia M. van Duijn, Andre G. Uitterlinden, Albert Hofman, Hannelore Steinböck, Ursula Gruber-Sedlmayr, Birgit Neophytou, Federico Zara, Andreas Hahn, Genetic Commission of the Italian League against Epilepsy, EuroEPINOMICS CoGIE Consortium, Padhraig Gormley, Felicitas Becker, Yvonne G. Weber, Maria Roberta Cilio, Wolfram S. Kunz, Roland Krause, Fritz Zimprich, Johannes R. Lemke, Peter Nürnberg, Thomas Sander, Holger Lerche, Bernd A. Neubauer

**Affiliations:** 1 Cologne Center for Genomics, University of Cologne, Cologne, Germany; 2 Psychiatric and Neurodevelopmental Genetics Unit, Massachusetts General Hospital and Harvard Medical School, Boston, Massachusetts, United States of America; 3 Program in Medical and Population Genetics, Broad Institute of MIT and Harvard, Cambridge, Massachusetts, United States of America; 4 Stanley Center for Psychiatric Research, Broad Institute of MIT and Harvard, Cambridge, Massachusetts, United States of America; 5 Department of Neurology, Medical University of Vienna, Vienna, Austria; 6 Institute of Biochemistry, Department of Chemistry, University of Cologne, Cologne, Germany; 7 Luxembourg Centre for Systems Biomedicine (LCSB), University of Luxembourg, Esch-sur-Alzette, Luxembourg; 8 Folkhälsan Institute of Genetics, Helsinki, Finland; 9 Neuroscience Center, University of Helsinki, Helsinki, Finland; 10 Research Programs Unit, Molecular Neurology, University of Helsinki, Helsinki, Finland; 11 CeGaT GmbH—Centre for Genomics and Transcriptomics, Tübingen, Germany; 12 Departments of Epidemiology, Neurology, Radiology, Erasmus Medical Center, Rotterdam, Netherlands; 13 Department of Epidemiology, Erasmus Medical Center, Rotterdam, Netherlands; 14 Department of Internal Medicine, Erasmus Medical Center, Rotterdam, Netherlands; 15 Private Practice for Pediatrics, Vienna, Austria; 16 Department of Pediatrics, Medical University of Graz, Graz, Austria; 17 St. Anna Children’s Hospital, Department of Neuropediatrics, Vienna, Austria; 18 Laboratory of Neurogenetics and Neuroscience, Institute G. Gaslini, Genova, Italy; 19 Department of Neuropediatrics, University Medical Center Giessen and Marburg, Giessen, Germany; 20 Department of Neurology and Epileptology, Hertie Institute for Clinical Brain Research, University of Tübingen, Tübingen, Germany; 21 Departments of Neurology and Pediatrics, University of California San Francisco, San Francisco, California, United States of America; 22 Department of Epileptology, University of Bonn, Bonn, Germany; 23 Institute of Human Genetics, University of Leipzig, Leipzig, Germany; 24 Cologne Excellence Cluster on Cellular Stress Responses in Aging-Associated Diseases (CECAD), University of Cologne, Cologne, Germany; Charité Universitätsmedizin Berlin, NeuroCure Clinical Research Center, GERMANY

## Abstract

**Objective:**

The *SCN1A* gene, coding for the voltage-gated Na^+^ channel alpha subunit Na_V_1.1, is the clinically most relevant epilepsy gene. With the advent of high-throughput next-generation sequencing, clinical laboratories are generating an ever-increasing catalogue of *SCN1A* variants. Variants are more likely to be classified as pathogenic if they have already been identified previously in a patient with epilepsy. Here, we critically re-evaluate the pathogenicity of this class of variants in a cohort of patients with common epilepsy syndromes and subsequently ask whether a significant fraction of benign variants have been misclassified as pathogenic.

**Methods:**

We screened a discovery cohort of 448 patients with a broad range of common genetic epilepsies and 734 controls for previously reported *SCN1A* mutations that were assumed to be disease causing. We re-evaluated the evidence for pathogenicity of the identified variants using *in silico* predictions, segregation, original reports, available functional data and assessment of allele frequencies in healthy individuals as well as in a follow up cohort of 777 patients.

**Results and Interpretation:**

We identified 8 known missense mutations, previously reported as pathogenic, in a total of 17 unrelated epilepsy patients (17/448; 3.80%). Our re-evaluation indicates that 7 out of these 8 variants (p.R27T; p.R28C; p.R542Q; p.R604H; p.T1250M; p.E1308D; p.R1928G; NP_001159435.1) are not pathogenic. Only the p.T1174S mutation may be considered as a genetic risk factor for epilepsy of small effect size based on the enrichment in patients (*P* = 6.60 x 10^−4^; OR = 0.32, fishers exact test), previous functional studies but incomplete penetrance. Thus, incorporation of previous studies in genetic counseling of *SCN1A* sequencing results is challenging and may produce incorrect conclusions.

## Introduction

The *SCN1A* gene (MIM#182389), coding for the voltage-gated Na+ channel alpha subunit NaV1.1, is the most clinically relevant epilepsy gene. *SCN1A* variants are associated, at the more benign end of the disease spectrum, with the dominantly inherited genetic epilepsy with febrile seizures plus [1, 2] (GEFS+), and, at the severe end, with Dravet syndrome (DS), an epileptic encephalopathy arising from *de novo SCN1A* mutations in the vast majority of DS patients [3]. More rarely, *SCN1A* mutations are also found in other types of infantile epileptic encephalopathies [4]. Common variants in *SCN1A* have been associated with mesial temporal lobe epilepsy and hippocampal sclerosis with febrile seizures in a genome-wide association study [5], and in a recent meta-analysis including 8696 patients with genetic generalized-, focal-, or unclassified epilepsies [6]. In around 88% of epilepsy patients carrying rare *SCN1A* mutations, these arise *de novo*, whereas only 12% of the affected individuals inherit the mutation from a, usually unaffected, parent [4]. Patients with an identical mutation may express a broad spectrum of phenotypes even within a single large family ranging from unaffected, over GEFS+ to Dravet syndrome [7, 8]. Most functional studies of disease-associated variants showed loss-of-function effects of *SCN1A* mutations [9–12]. Mutations with a complete loss of function, in general, lead to more severe epilepsies [3, 13, 14].

Massive parallel sequencing studies have accelerated mutation discovery. With declining costs, more patients will be sequenced, and many newly identified mutations are expected. Determination of the functional consequences and pathogenicity is challenging and molecular studies for each variant are currently not feasible. Databases like the "The Human Gene Mutation Database" (HGMD; http://www.hgmd.org/) constitute a comprehensive collection of mutations in genes underlying or associated with human inherited diseases. The database is routinely accessed and utilized by next generation sequencing (NGS) project researchers, human molecular geneticists, neurologists and genetic counselors.

Interpretation of genetic results is challenging, especially in multifactorial diseases like common epilepsies. The HGMD comprises more than 1000 disease-associated mutations annotated for the *SCN1A* gene (accessed Dec/2014). Here, we investigated a already whole-exome sequenced cohort of 448 patients with a broad range of common genetic epilepsies, for SCN1A mutations listed as disease associated in the HGMD. We re-evaluated the identified variants’ evidence for pathogenicity by *in silico* prediction, segregation, literature review for clinical, genetic and functional relevance and assessment of allele frequencies in healthy individuals. Finally, we investigated the phenotypic spectrum of the most reliable variant in a diagnostic epilepsy cohort.

## Patients and Methods

### Patients and sequencing

Patients of European and Turkish ancestry were recruited at several centers from Germany, Austria, Italy, Finland, Canada and Turkey. We included Turkish patients since *SCN1A* variant frequencies do not show correlation with the ethnicity when compared between Asian and European patients [4]. Samples from The Rotterdam Study [15] not specifically screened for European ethnicity, which were handled in the same way as our cases, i.e. using the same enrichment and sequencing methods, served as controls for the whole exome dataset. The controls are drawn from the population and are not evaluated for seizure disorders. Informed consent for whole exome sequencing was obtained from all participants or legal representatives respectively. The two institutions in charge of the reported analysis are the University hospitals in Tübingen (for “genetic generalized epilepsy”) and Giessen (for “genetic focal epilepsy”), Germany. Both review boards in Tübingen and Giessen approved this study.

High-throughput, targeted sequencing was performed as previously described [16] with the Nimblegen-SeqCapEZ-V244M enrichment kit on the Illumina HiSeq2000 system. For each sample, we calculated the gene-coverage of *SCN1A*. Only samples with a minimum of 90% of all bases in the coding region of the gene (i.e. all exons plus the first and last five intronic bases, NM_001165963.1) being covered by at least 15 reads were used. After quality control, 448 unrelated epilepsy patients (235 with genetic generalized epilepsy; 182 with rolandic epilepsy; 22 with atypical rolandic epilepsy; 4 with benign familial neonatal seizures; 4 with benign familial infantile epilepsy; 1 with benign adult familial myoclonic epilepsy; 226 males, 222 females) and 734 control individuals (435 females, 299 males) were included in the analysis. All *SCN1A* HGMD variants identified in the patients were validated with Sanger sequencing using standard protocols. Primers are available upon request. Rare *SCN1A* sequence variants, which are not annotated by HGMD, were not investigated in this study.

### HGMD annotation and assessment of the literature

Variant annotation was performed using the HGMD Professional 2013.4–15th December 2013 Version [17]. We assessed original reports cited by the HGMD for each identified HGMD patient mutation. We re-analyzed the mutations, and if the original report used the sequence of a shorter transcript of the *SCN1A* gene for numbering we numbered the variant accordingly to the RefSeqGene (NM_001165963.1; which represent the longest transcript and reference protein NP_001159435.1) used in our data set.

### Assessment of mutation frequency in controls

Besides our control dataset, variant frequencies were taken from the ExAC collection (http://exac.broadinstitute.org/). Note that this database also includes patients from the "Swedish Schizophrenia & Bipolar Studies”. Furthermore, it is not clear if patients with mild seizure disorders have been excluded.

### In silico prediction

Functional prediction scores were obtained from the dbNSFP database version 2.8 (http://sites.google.com/site/jpopgen/dbNSFP, accessed 01/2015). In total we used six prediction scores (SIFT, Polyphen-2-HVAR, Polyphen-2-HDIV, Mutation Assessor, FATHMM, LRT) and two conservation scores (GERP++, PhyloP). We did not use the dbNSFP generated ensemble scores, as they integrate the prediction of the tool Mutation Taster, which itself incorporates HMGD entries. We classified the variant as “damaging” when the majority of the tools predicted a functional effect for the variant (i.e. a minimum of 5 out of 8 tools). Topology and domain structure were taken from UniProt entry P35498.

### Screening of the p.T1174S mutation in a diagnostic cohort of epilepsy

To further investigate the frequency and phenotypic spectrum of the p.T1174S mutation, we evaluated NGS panel sequencing data of epilepsy-associated genes [18] of 777 individuals provided by the CeGaT diagnostic lab (http://www.cegat.de, genes listed in S1 Table). Patients covered a broad spectrum of epilepsy phenotypes, often associated with intellectual disability.

### Criteria for variant re-classification

We consider a variant as pathogenic if all of the following three criteria are met: i) The variant is statistically enriched in the patient cohort and/or absent in controls; ii) the segregation pattern analysis and the re-evaluation of original reports do not weaken the epilepsy association, iii) the variant is predicted to be pathogenic by the majority of *in silico* classifiers and/or molecular functional analyses supports its pathogenicity.

## Results

### Case vs. control HGMD analysis

We analyzed sequence data of 448 epilepsy patients and 734 controls for *SCN1A* variants previously reported as disease causing collected by the HGMD. Our mutation screening identified eight distinct HGMD missense mutations (Table 1: p.R27T, p.R28C, p.R604H, p.R542Q, p.T1174S, p.T1250M, p.E1308D, p.R1928G) affecting in total 17 unrelated epilepsy patients (17/448 = 3.80%) compared to six HGMD variants affecting 10 control individuals (10/734 = 1.36%). None of these patients were carrier of a rare loss of function variant in a known epilepsy gene (S2 Table). The *SCN1A* HGMD variant frequency was therefore slightly enriched in the patient cohort (*P* = 8.30 x 10^−3^). A high allele frequency in public databases would argue against pathogenicity of the identified *SCN1A* HGMD variants identified in our patients. In addition to our own sequenced controls we investigated the *SCN1A* HGMD variant frequencies further using the ExAc, a publicly accessible variant database of 60706 unrelated individuals. All eight variants were found in individuals collected in the ExAc database (Table 1). After combining our in-house control and ExAc data in a meta-analysis, only the p.T1174S variant remained significantly enriched after correction for multiple testing (Table 1) in epilepsy patients supporting p.T1174S as the only identified *SCN1A* genetic risk factor for epilepsy in our cohort.

**Table 1:** HGMD *SCN1A* mutation frequencies in patients and controls.

Mutation	dbSNP ID	Patient mutation carrier in(N = 448)	Controls (N = 734)	Patient vs. Controls	ExAC (N = 60706)	Patient vs. controls + ExAC
p.R1928G	rs121917956	1	0	-	171/60605	1
p.E1308D	rs121917910	1	2	-	91/60276	0.498
p.T1250M	rs140731963	1	0	-	42/58362	0.277
p.T1174S	rs121918799	6	0	**3.02 x 10**^**−3**^	214/60675	**5.61 x 10**^**−3**^
p.R604H	rs121918769	5	1	7.91 x 10^−3^	185/60690	0.013
p.R542Q	rs121918817	1	5	-	186/60687	1
p.R28C	No ID	1	0	-	4/60701	0.036
p.R27T	rs121917906	1	0	-	14/60702	0.103

Accession according to reference transcript NM_001165963.1; and references protein NP_001159435.1; P values ware calculated either by Fisher’s exact test; bold P values are still significant after Bonferroni correction = α/n. For testing eight mutations with a desired α = 0.05, the Bonferroni correction tests each individual hypothesis at α = 0.05/8 = 0.00625

### Segregation analysis

**Fig 1:**
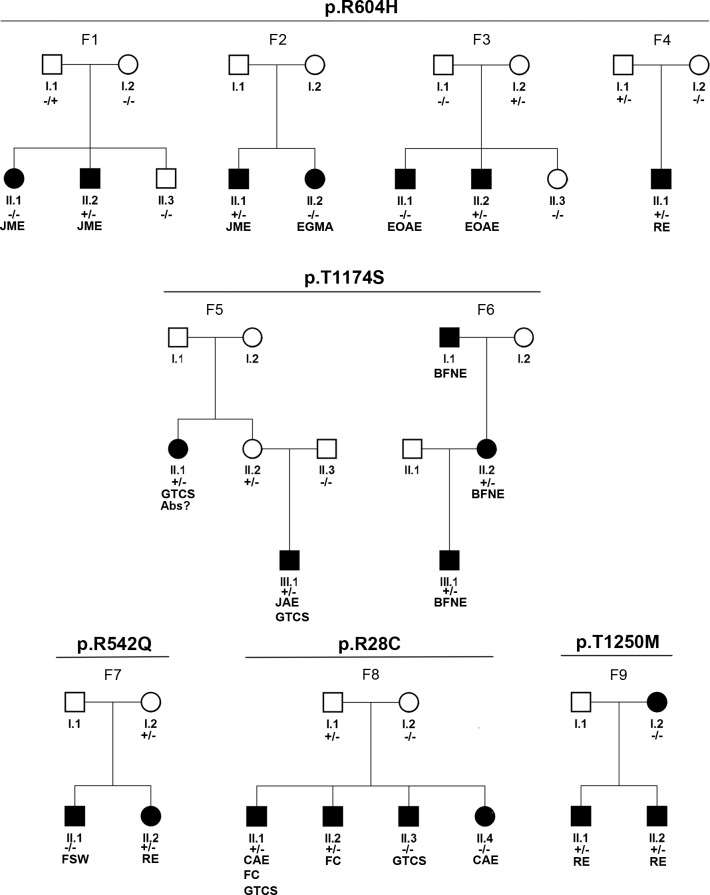
Segregation analysis of the identified variants. Analysis of likely segregation of the respective mutations in family members could be performed in eleven families. Accession is according to reference transcript NM_001165963.1; and references protein NP_001159435.1. Abbreviations: FC = febrile convulsion; JME = juvenile myoclonic epilepsy, CAE: childhood absence epilepsy, JAE: juvenile absence epilepsy, EGMA: epilepsy with generalized tonic-clonic seizures predominantly on awakening, EOAE = Early onset absence epilepsy, RE = Rolandic epilepsy, Abs = absence, BFNE = Benign familial neonatal seizure, FSW = Focal Sharp Waves, GTCS = Generalized tonic-clonic seizure

We further analyzed the family segregation pattern whereby a shared segregation of the *SCN1A* HGMD variant and epilepsy was evaluated as support for the variant pathogenicity. For five variants detected in patients (p.T1250M, p.T1174S, p.R604H, p.R542Q, p.R28C), segregation analysis was possible in a total of nine families (Fig 1). All variants were inherited, five times maternally and three times paternally, six times from a healthy parent and only twice from an affected parent. Only the p.T1174S variant segregated with epilepsy in our investigated families (Fig 1).

### Review of the original reports

Next, we went back to the 16 original reports (1–4 cited reports for each mutation, Table 2) of all eight identified patient mutations referenced in the HGMD to summarize inheritance, segregation and available functional data. The majority of the reports do not support the disease association of the variant from a today’s perspective. None of the mutations have been described as *de novo*. In three reports (p.R604H, p.E1308D, p.R1982G) an additional *SCN1A* mutation has been reported in the HGMD referenced patient, including a stop codon mutation (p.R604H in combination with p.R1525X; p.R1928G in combination with p.L1207P, and p.E1308D in combination with p.A239V; Table 2). In another study the HGMD referred patient had two additional *SCN1A* mutations, one missense and one splice site acceptor variant (Table 2, p.T1250M in combination with p.R27T and c.3706-2A > G). In four families the mutation was inherited (each one family: p.R27T, p.R542Q, p.T1174S, p.E1308D; Table 2) from a healthy parent and segregation was not determined in 12 families. In contrast, only in three families the mutation segregated with the disease, one affected by the p.R604H and two by the p.T1174S variants. Furthermore, only for the p.T1174S variant functional studies have been conducted. In particular, experiments in tsA-201 cells and computational modeling of mutant properties both supported its epileptogenic effect [19].

**Table 2:** Summary of previous studies referenced at the HGMD specific for each identified variant.

Mutation	Previous Phenotype	Previous inheritance	Comment	Ref	Phenotypes in this study
p.R1928G	GEFS+	Not determined	-	[1]	1x CAE
	Control	Not determined	-	[1]	
	SMEI	Not determined	Patient carried an additional p.L1207P mutation in *SCN1A*	[20]	
p.E1308D	FC	Not determined	-	[21]	1x JAE
	SMEI	Inherited from healthy father	Patient carried an additional m p.A239V mutation in *SCN1A*	[22]	
p.T1250M	DS	Not determined	Patient has an additional SCN1A missense mutation (p.Arg27Thr) and one variant predicted as splice Site acceptor mutation (c.3706-2A > G)	[23]	1x RE
	GEFS+		-	[21]	
p.T1174S	DS + Migraine	Maternal inherited	Patient has DS, mother has migraine with aura	[24]	1x GTCS, 2x RE, 1x
	SMEI	Not determined	-	[25]	BFNE, 1x CAE, 1x JAE
	Seizures and hemiplegic migraine	Large family: all mutation carriers had epilepsy or migraine. 3x migraine with aura, 1 migraine without aura and benign occipital epilepsy, 2x migraine with aura and benign occipital epilepsy = > inherited	Functional studies, interpretation = modulation of the properties of T1174S can lead to a switch between overall gain and loss of function, consistent with a switch between promigraine end epileptogenic effect and, thus, with coexistence of epileptic and FHM phenotypes in the same family.	[19]	
	MAE	Inherited from healthy mother	-	[26]	
	FHM	Not determined	-	[27]	
p.R604H	Intractable Epilepsy	Not determined		[28]	2x JME;
	FS, FS+	Inherited all affected	With a single family, All three mutation carrier had FS or FS+	[29]	1x CAE, 1xRE, 1x EOAE
	DS	Not determined	Patient carries also a *SCN1A* Stop codon mutation (p.R1525X)	[30]	
p.R542Q	JME	Not determined	-	[31]	1x RE
	Familial autism	Inherited from healthy father	A sib with autism is affected and carries the mutation as well	[32]	
	ICE	Not determined	-	[28]	
p.R28C	GEFS+	Not determined	-	[33]	1x JAE
p.R27T	GEFS+	Inherited from healthy father	-	[22]	1x JME

Abbreviations: GEFS+ = genetic epilepsy with febrile seizures plus; SMEI = severe myoclonic epilepsy of infancy; FC = febrile convulsion; DS = Dravet Syndrome; MAE = Myoclonic astatic epilepsy (Doose syndrome); FHM = Familial hemiplegic migraine; FS = febrile seizures; JME = juvenile myoclonic epilepsy, ICE = Intracable childhood epilepsy; GGE syndromes: CAE: childhood absence epilepsy, JAE: juvenile absence epilepsy, JME: juvenile myoclonic epilepsy, EGMA: epilepsy with generalized tonic-clonic seizures predominantly on awakening, EGTCS: epilepsy with generalized tonic-clonic seizure

### In silico prediction analysis

Six of the eight identified variants (p.R28C, p.R542Q, p.R604H, p.T1250M, p.E1308D and p.R1928G) are predicted to have a damaging effect when both structural prediction and local conservation scores were considered (Table 3). In contrast to previous molecular studies [19] and our statistical enrichment and segregation studies, the p.T1174S variant was not predicted to be damaging by the majority of prediction tools (Table 3).

**Table 3:** Functional prediction and conservation scores of the 8 HMGD Variations.

Mutation	SIFT	Polyphen2_HDIV	Polyphen2_HVAR	LRT_pred	MutationAssessor	FATHMM	GERP++ rankscore	PhyloP46way_primate rankscore	predicted outcome (n of tools)
p.R27T	T	B	B	N	L	D	0.589	0.943	functionally neutral (3/8)
p.R28C	D	P	B	D	M	D	0.912	0.943	damaging (7/8)
p.R542Q	D	B	B	D	L	D	0.997	0.943	damaging (5/8)
p.R604H	T	D	D	D	M	D	0.772	0.741	damaging (7/8)
p.T1174S	T	B	B	N	N	D	0.789	0.430	functionally neutral (2/8)
p.T1250M	T	D	B	D	M	D	0.534	0.711	damaging (6/8)
p.E1308D	T	D	P	D	N	D	0.802	0.533	damaging (6/8)
p.R1928G	T	B	B	D	M	D	0.638	0.697	damaging (5/8)

T = tolerated, D = damaging, B = benign, P = possibly damaging, N = neutral, L = predicted non-functional low, M = predicted functional medium, N = predicted non-functional neutral. For GERP++ and PhyloP rankscores a cutoff of 0.5 was used in order to rank the variant site as conserved. The variant was classified as “damaging” when the majority of tools predicted a functional effect (damaging, possibly damaging, predicted functional-damaging) and classified as „functionally neutral”when the majority of the tools predicted no functional effect (neutral, tolerated, benign, non-functional).

### Replication analysis p.T1174S

Overall, after the initial re-evaluation of the identified *SCN1A* HGMD variants, we suggest that only the p.T1174S variant is potentially a epilepsy associated variant. The p.T1174S variant is enriched in patients, segregates with the phenotype in families and published functional data support pathogenicity of the variant (Table 2). A major factor in our variant classification is comparing the allele frequency differences between patients and controls. To further assess the frequency of p.T1174S alleles in epilepsy patients and replicate our association, we investigated an additional epilepsy cohort provided by the CeGaT diagnostic lab using targeted sequencing. We identified seven out of 777 epilepsy patients (0.90%) carrying the p.T1174S *SCN1A* mutation. Four of the patients were diagnosed with intellectual disability and epilepsy, one patient had temporal lobe epilepsy, and two patients had epileptic seizures without further specification of the phenotype. In five of the seven patients, the mutation had been inherited from a healthy parent. In the remaining two cases, parents were not available for segregation testing. Of note, in one of the seven patients, an additional pathogenic variant was detected by detection of a *de novo* mutation in *GRIN2B* [34] (c.1619G>A, p.R540H).

Combining both epilepsy cohorts together, we have identified 13 out of 1219 patients carrying the p.T1174S mutation. A comparison of all available patient and control data confirms enrichment in epilepsy patients (*P* = 5.68 x 10^−4^; OR = 3.08, 95%-CI: 1.61–5.40, fisher exact test, 13/1219 patients vs. 21461409 controls).

## Discussion

In the context of molecular genetic testing, it is often challenging to establish the pathogenicity of an intragenic variant. Lack of parental DNA or control individuals, absence of functional data and association with different phenotypes add to this complexity.

In this study, we investigated the liability of *SCN1A* variants that were previously classified as pathogenic, in a cohort of common epilepsy syndromes. We detected an enrichment of HGMD annotated variants in our epilepsy patient cohort compared to controls (*P* = 8.30 x 10^−3^). All identified HGMD *SCN1A* variants in our patients have also been detected in control individuals arguing against completely penetrant causal mutations. None of the variants were localized in the transmembrane regions of the protein (S1 Fig), where mutations are associated with more severe channel dysfunctions [13]. We considered seven, out of the eight (p.R27T, p.R28C, p.R542Q, p.R604H p.T1250M, p.E1308D, p.R1928G) analyzed HGMD variants, as obvious benign based on the original reports, the high frequency in control individuals and missing or negative segregation and functional results (Tables 1 and 2; Fig 1).

In contrast to the other mutations classification of the p.T1174S mutation is less trivial. On the one hand, support as a potential genetic risk factor for epilepsy is based on several reasons. Firstly, the p.T1174S variant is overrepresented in epilepsy patients (*P* = 5.68 x 10^−4^). Secondly, it segregates with the disease in our, and, previously analyzed families (Table 2, Fig 1). It was previously described in patients diagnosed with severe myoclonic epilepsy of infancy (inheritance not determined), Dravet syndrome (inherited from a mother with migraine), myoclonic astatic epilepsy (inherited from a healthy mother), and repetitively with familial hemiplegic migraine (FHM) [24, 27]. In our cohort, the p.T1174S mutation was found in patients with rolandic epilepsy, childhood absence epilepsy, juvenile absence epilepsy, benign familial neonatal seizures epilepsy and patients with a single generalized tonic-clonic seizure. In the diagnostic cohort we found patients with unclassified epilepsy with intellectual disability as well as temporal lobe epilepsy. Additionally, another family with the p.T1174S mutation has been identified (personal communication with Arvid Suls, University of Antwerp). In this family the mother carries the variant, as do the six elder siblings of seven. Two of these six carriers are affected by epilepsy with myoclonic seizures. Finally, previous *in vitro* electrophysiological recordings of the mutant p.T1174S Nav1.1 channel support the variant pathogenicity [19]. The molecular analysis revealed two divergent effects: a positive shift of the activation curve and deceleration of recovery from fast inactivation–consistent with a loss of function, and an increase of persistent current (I(NaP))–consistent with a gain of function [19]. On the other hand, the p.T1174S variant was only predicted to be damaging by one *in silico* program (FATHMM, Table 3**)** and was inherited by unaffected parent in the replication cohort five out of seven times.

Besides the unclear role of the p.T1174S variant, the majority of our investigated *SCN1A* HGMD variants cannot be classified as clearly pathogenic. Based on our results, we assume that a significant fraction of patients diagnosed with pathogenic *SCN1A* mutations may actually not carry an *SCN1A* variant of relevance. The role of *SCN1A* missense mutations in the pathogenesis of common epilepsies may thus be overstated (in general and e. g. studies in Table 2). Previous conclusions were frequently based on comparisons of allele frequencies between patients and small control datasets often without molecular follow up of the variant (Table 2). By accessing large gene mutation databases of non-epileptic individuals (e.g. http://exac.broadinstitute.org) we show that previous studies have been underpowered. In order to draw a definitive conclusion about pathogenicity for variants in common epilepsy syndromes, which are also present in healthy individuals, functional studies are mandatory (e.g. functional characterization of patient neurons derived form induced pluripotent cells).

The conclusions drawn from our study can be translated to other fields of research. Several prediction tools [35–39] evaluating the pathogenic potential of DNA sequence alterations are trained with variant lists extracted from the HGMD. Given that the disease association of the annotated *SCN1A* variants have to be questioned, it is likely that a similar picture can be seen for other complex diseases and genes. Simply extracting HGMD variants as training sets might bias prediction algorithms and machine learning approaches.

Overall, we highlight the ambiguities of variant classification in common epilepsy syndromes and emphasize that the majority of *SCN1A* variants could not be re-classified as pathogenic. Furthermore, our results warrant careful assessment of variants previously reported in small cohort studies.

## Supporting Information

S1 Fig SCN1A domain organization and mutations. The schematic diagram showing the domain organization of the alpha subunit of the voltage-gated sodium ion channel coded by the SCN1A gene and the positions of the missense mutations (shown as orange circles). The complex consists of four homologous domains (I-IV), each containing six transmembrane segments (S1-S6). IQ indicates the localization of the IQ calmodulin-binding motif.
(PDF)

S1 Table List of genes included in the gene panel. (XLSX)

S2 Table Rare loss of function variants in 17 epilepsy patients with known HGMD SCN1A variants. (XLSX)
