# Supplementary figures and images for: Evaluation of Presumably Disease Causing SCN1A Variants in a Cohort of Common Epilepsy Syndromes
Source: PLoS One. 2016 Mar 18;11(3):e0150426. doi: 10.1371/journal.pone.0150426 (PMC4798642; doi:10.1371/journal.pone.0150426)

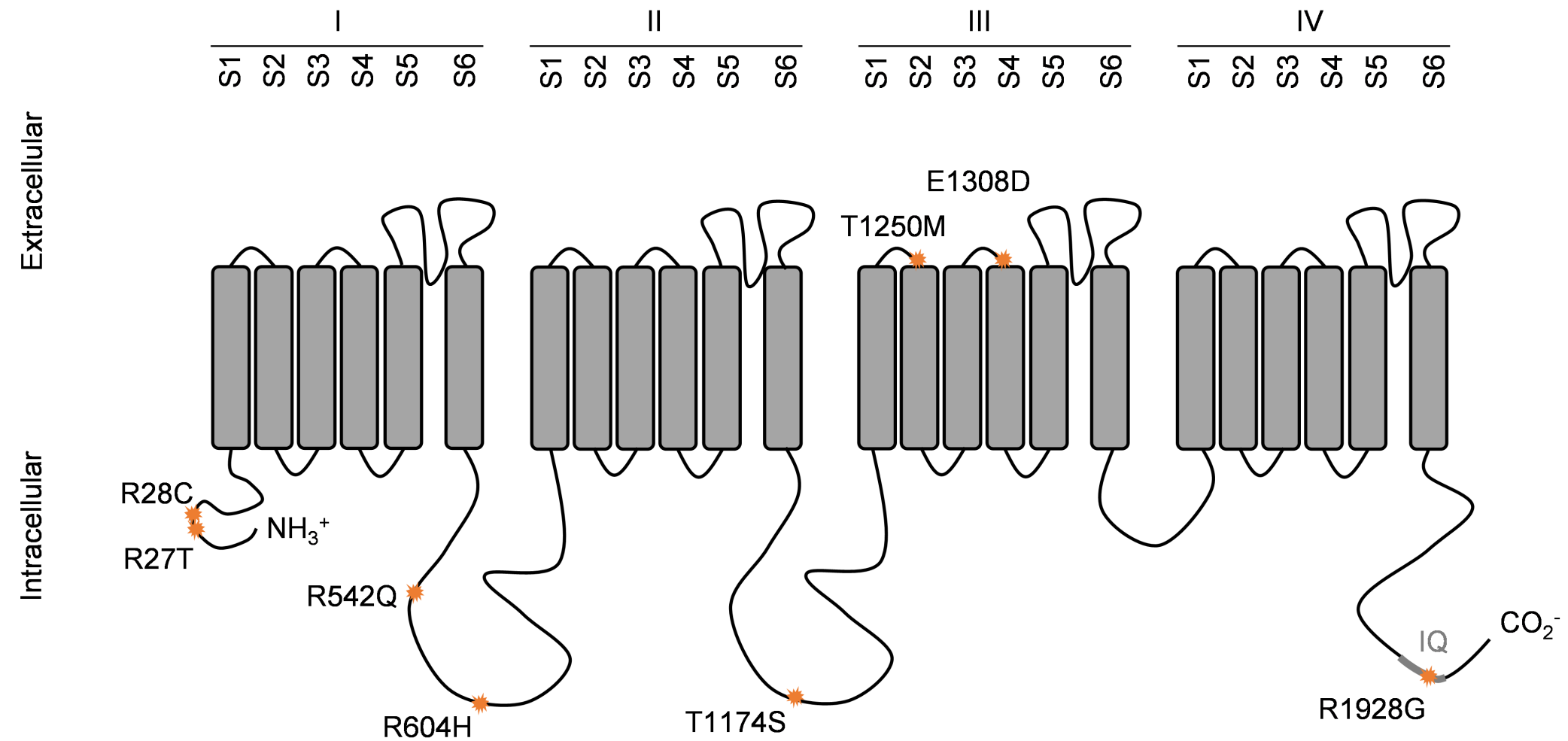

Supplement: S1 Fig — The schematic diagram showing the domain organization of the alpha subunit of the voltage-gated sodium ion channel coded by the SCN1A gene and the positions of the missense mutations (shown as orange circles). The complex consists of four homologous domains (I-IV), each containing six transmembrane segments (S1-S6). IQ indicates the localization of the IQ calmodulin-binding motif. (PDF) [file pone.0150426.s001.pdf]
